# New Insights into Nanoplastics Ecotoxicology: Effects of Long-Term Polystyrene Nanoparticles Exposure on *Folsomia candida*

**DOI:** 10.3390/toxics11100876

**Published:** 2023-10-22

**Authors:** Angela Barreto, Joana Santos, Gonçalo Andrade, Matilde Santos, Vera L. Maria

**Affiliations:** Department of Biology & Centre for Environmental and Marine Studies (CESAM), University of Aveiro, Campus of Santiago, 3810-193 Aveiro, Portugal

**Keywords:** collembola, multigenerational impact, oxidative damage, plastics, terrestrial environment

## Abstract

Despite the growing concern over nanoplastics’ (NPls) environmental impacts, their long-term effects on terrestrial organisms remain poorly understood. The main aim of this study was to assess how NPls exposure impacts both the parental (F1) and subsequent generations (F2 and F3) of the soil-dwelling species *Folsomia candida*. After a standard exposure (28 days), we conducted a multigenerational study along three generations (84 days), applying polystyrene nanoparticles (PS NPs; diameter of 44 nm) as representatives of NPls. Endpoints from biochemical to individual levels were assessed. The standard test: PS NPs (0.015 to 900 mg/kg) had no effect in *F. candida* survival or reproduction. The multigenerational test: PS NPs (1.5 and 300 mg/kg) induced no effects on *F. candida* survival and reproduction along the three generations (F1 to F3). PS NPs induced no effects in catalase, glutathione reductase, glutathione S-transferases, and acetylcholinesterase activities for the juveniles of the F1 to F3. Oxidative damage through lipid peroxidation was detected in the offspring of F1 but not in the juveniles of F2 and F3. Our findings underscore the importance of evaluating multigenerational effects to gain comprehensive insights into the contaminants long-term impact, particularly when organisms are continuously exposed, as is the case with NPls.

## 1. Introduction

The widespread use of plastics in our daily lives has led to the accumulation of plastic waste in the environment, including terrestrial ecosystems [1]. Interacting with the natural environment, large plastics undergo degradation through physical, chemical, and biological processes (e.g., mechanical forces, photodegradation, UV degradation, and biodegradation) [2]. Plastics breakdown results in smaller particles, such as nanoplastics (NPls), which exhibit colloidal properties in aqueous systems and range in size from 1 up to 1000 nm [3].

NPls are persistent pollutants that accumulate in the environment for extended periods due to their extremely poor degradability [4]. Moreover, their small sizes allow them to be taken up by various organisms, raising concerns about potential bioaccumulation and biomagnification [5]. This poses a threat to the health and survival of terrestrial species. In terrestrial environments, such as soils, the quantity of mismanaged plastic waste can be up to 4–23 times greater than in marine ecosystems [6,7]. Therefore, concerns regarding the environmental impacts of NPls on terrestrial environments have been growing. However, information about the long-term effects (over multiple generations) of NPls exposure on the health and viability of terrestrial organisms and the mechanisms by which NPls may impact ecosystem dynamics and function remain poorly understood [8]. Therefore, further research is needed to answer these questions as organisms, along their life stages, their offspring, and subsequent generations, are constantly exposed to plastics. Obtaining this information is urgent to develop effective strategies for mitigating NPls’ impacts and protecting the health and sustainability of these vital systems. 

Assessing multigenerational effects allows us to understand the full impact of contaminants on populations over time as many contaminants can have long-lasting effects that are not immediately apparent [9]. Indeed, some studies have reported no significant effects on the survival and reproduction of terrestrial invertebrate species such as *Folsomia candida* [10] and *Enchytraeus crypticus* [11] after 28 and 21 days (d) NPls exposure, respectively. Therefore, it is essential to determine whether the absence of effects observed in a standard reproduction test persists through subsequent generations. 

Based on this, the main research question of the current study is as follows: how does exposure to NPls impact the parental generation and the subsequent generations of *F. candida*? We conducted a multigenerational study over three generations (F1-parental to F3; time: 84 d) using *F. candida* as terrestrial model organism and polystyrene nanoparticles (PS NPs; diameter of 44 nm) as representative of NPls. We assessed endpoints ranging from biochemical (neurotransmission, antioxidant, and oxidative damage processes) to individual (survival and reproduction) levels in all the generations.

*F. candida* has a worldwide distribution and is a detritivore arthropod that inhabits the upper soil profile [9]. According to Guimarães et al.’s (2023) review, 16 studies have successfully employed *F. candida* for multigenerational studies assessing the effects of different types of contaminants (organic, metals, or others) [9]. Concerning multigenerational tests using PS NPs, we found only five studies with invertebrates, such as *Daphnia magna* (freshwater; [12,13]) *Brachionus plicatilis* (seawater; [14]), and *Caenorhabditis elegans* (soil pore water; [15,16]). These studies showed dissimilar results, with PS NPs inducing no adverse multigenerational effects for *D. magna* in terms of survival, reproductive endpoints, body length (adults and offspring), and lipid content (offspring) [12]. In contrast, PS NPs caused severe multigenerational life-history trait changes (NPls suppressed population growth, negatively affected life span, the first spawning and breeding time, and fecundity) and metabolic responses (disruption on purine-pyrimidine metabolism, tricarboxylic acid cycle, and protein synthesis pathway) in *B. plicatilis* [14]. PS NPs also led to multi- and trans-generational reproduction decline that was associated with germline toxicity and epigenetic regulation in *C. elegans* [15]. Our current research represents a significant advancement in this field as it provides novel insights into the multigenerational effects of NPls on *F. candida*, spanning from the F1 to F3 generations. Given the limited and diverse nature of the available findings on NPls’ multigenerational impacts, our study underscores its importance and contributes valuable data to this emerging area of research.

Our hypothesis is that the toxic effects of PS NPs increase over the time of exposure, with long-lasting effects being detected in the F2 and/or F3 generations, but not in the parental generation. Moreover, effects at a biochemical level will likely be found earlier than individual responses, as described in a previous study with *F. candida* where no PS NPs effects were found on reproduction and survival (28 d exposure), but lipid peroxidation (LPO) induction was observed in the offspring [10].

## 2. Materials and Methods

### 2.1. Test Species

The standard species *F. candida* (Collembola), a terrestrial model organism in ecotoxicology [17], was chosen for the toxicity tests. Organisms’ cultures were kept in laboratory, on a moist substrate of plaster of Paris and activated charcoal (8:1 ratio), at 20 ± 1 °C, under a photoperiod of 16 hours (h): 8 h (light: dark). The organisms were fed weekly with dried baker’s yeast (*Saccharomyces cerevisae*). To start the tests, organisms from these cultures were age synchronized to obtain juveniles with 10–12 d.

### 2.2. Test Medium

The natural standard LUFA 2.2 soil (Speyer, Germany) was applied as test medium. According to the provider information, the primary characteristics included a pH of 5.6, organic carbon content at 1.71%, cation exchange capacity measuring 9.2 meq/100 g, a maximum water-holding capacity (WHC) of 44.8%, and a grain size composition consisting of 8.9% clay, 13.9% silt, and 77.2% sand. The soil was subjected to a drying process (48 h; 60 °C) prior to its use. 

### 2.3. Test Contaminant

PS NPs stock dispersion was purchased from Polysciences Europe GmbH, Germany (Brand: Bangs Laboratories, Inc., Fishers, IN, USA). More information about this dispersion is available in the Supplementary Material. PS NPs’ stock dispersion was centrifuged before the toxicity tests using a Vivaspin^®^ 2 mL ultrafiltration device (Bangs Laboratories, Inc., Fishers, IN, USA) to remove sodium dodecyl sulfate and sodium azide existent in the dispersion. The PS NPs’ stock dispersion (centrifuged, prepared in ultrapure water) and aqueous test dispersions used for multigenerational test (correspondent to 1.5 and 300 mg/kg) were characterized by hydrodynamic size (HS), evaluated by dynamic light scattering (Zetasizer Nano ZS, Malvern, PA, USA) and by zeta potential (ZP), assessed by electrophoretic light scattering (Zetasizer Nano ZS, Malvern, PA, USA). The Zetasizer Nano ZS (Malvern, PA, USA) was also allowed to obtain the polydispersity index (PdI) of the PS NPs dispersions. More details in the Supplementary Material.

### 2.4. Toxicity Tests

#### 2.4.1. Soil Spiking Procedures

A full concentration range of PS NPs was tested: 0–0.015–15–150–600–900 mg/kg soil dry weight (DW). This range was already tested in a previous study with other soil species (*E. crypticus*) showing no PS NPs’ effects in organisms’ survival and reproduction [11]. In terms of environmentally relevant concentrations, polystyrene NPls and microplastics’ concentration of 8.56 ± 0.04 mg/kg was quantified in a river sediment [18]. The control soil (0 mg/kg of PS NPs) was made with deionized water considering the adequate moisture content (50% of the maximum WHC). For PS NPs’ experimental conditions, the needed volumes of PS NPs test dispersions (prepared in ultrapure water) were added to the pre-moistened soil (in which water was added before) until 50% of the WHC maximum and mixed manually. The replicates were mixed individually. Toxicity tests started 1 d after soil spiking.

#### 2.4.2. Standard Reproduction Test

The experimental protocols conformed to the established Collembolan Reproduction Test in Soil guidelines [19], with certain modifications. In summary, each test vessel contained 10 age-synchronized individuals (10–12 d) and 30 g of moist soil along with a food source (dried baker’s yeast). The testing environment maintained a temperature of 20 ± 1 °C and followed a 16 h: 8 h photoperiod. The test ran for 28 d and food (10 mg) and water loss (based on the lost weight—all the vessels were weighed at the beginning and weekly during test development) was replenished weekly, as described in OECD 232 [19]. Four replicates per experimental condition (n = 4) were applied. An extra replicate per condition (not including organisms) was made to the measurement of the pH values.

On the 28th day, water was introduced into each test container, and the contents were subsequently transferred to a crystallizer dish. The crystallizer dish surface was then captured in photographs for subsequent organism counting using ImageJ software (version 1.54d). We evaluated survival, which involved counting the number of adult organisms, and reproduction, which entailed determining the number of juveniles. Further details on the organism counting process are available in the Supplementary Material. 

#### 2.4.3. Multigenerational Test (F1 to F3)

Based on the results obtained from the standard reproduction test, a multigenerational test, considering three consecutive 28 d exposures involving F1–F3 generations, was performed. The test was conducted following the same OECD guideline [19] as for reproduction test, except that at the test end some of the juveniles were sampled for biochemical analysis and some were further exposed to continue the multigenerational test. The following concentrations were tested: 0, 1.5 and 300 mg PS NPs/kg soil DW. Upon concluding the test, the flooding and photographing procedures were completed for organisms counting, utilizing the functions provided by the ImageJ software. Subsequently, the juveniles were transferred using a sieve to a container with a layer of plaster of Paris (culture medium). To expose the next generation, we selected the 10 largest juveniles, approximately 11 d old, and placed them in new test containers with freshly spiked soil. In addition, we collected 300 juveniles per replicate, which were then placed in microtubes and promptly frozen in liquid nitrogen. These samples were stored at −80 °C for subsequent biochemical analysis. This entire process was replicated for all 3 generations, comprising exposures of 28, 56, and 84 d for each successive generation of juvenile collembolans. The endpoints evaluated were survival and reproductive output.

### 2.5. Biochemical Markers Analysis

Procedures followed the previously optimized protocols by Maria et al. (2014) [20] for *F. candida*, with some adaptations (in terms of organism number used per replicate and organisms’ age). Catalase (CAT), glutathione reductase (GR), glutathione S-transferases (GST), acetylcholinesterase (AChE) activities and LPO levels were assessed for the juveniles resultant from F1 to F3 generations. Protein concentration was determined using bovine γ-globuline as a standard [21]. For CAT, GR, and GST activities, Clairborne (1985) [22], Carlberg, and Mannervik (1975) [23] and Habig et al.’s (1974) [24] methods were followed, respectively. LPO levels were measured according to Ohkawa et al. (1979) [25] and Bird and Draper (1984) [26], adapted by Filho et al. (2001) [27]. AChE activity was quantified according to Ellman et al. (1961) [28], adapted by Guilhermino et al. (1996) [29].

### 2.6. Data Analysis

Graphics and statistics assessment were performed using the Sigma Plot 12.5 software package (Munich, Germany). Shapiro–Wilk test to examine data for normality and Levene’s test to assess homoscedasticity were applied. To evaluate differences between the control group and PS NPs treatments, a one-way ANOVA (Analysis Of Variance) was used and, subsequently, a Dunnett’s multiple comparison post hoc test was employed. In cases where data did not meet the normality and homoscedasticity criteria, we opted for a non-parametric Kruskal–Wallis test. Significance was established at a significance level (*p*) < 0.05.

## 3. Results and Discussion

Despite the predicted plastic release into soil being approximately 40 times higher than into aquatic ecosystems [30], the number of ecotoxicity studies with microplastics and NPls in terrestrial environments is considerably low (8%) compared with aquatic ecosystems (92%: marine (61%) and freshwater (31%)) [8]. If we only consider research specifically focused on NPls, the number of studies is even lower, despite previous evidence demonstrating that NPls exert more pronounced impairments compared to their micro-sized counterparts [14,31,32].

### 3.1. Characterization of Polystyrene Nanoparticles

The PS NPs’ stock dispersion, after centrifugation, presented the expected HS (47.5 ± 0.1 nm) with a lower PdI (0.2) (Figure S1). In terms of ZP, the PS NPs’ stock dispersion presented a negative value (−23.6 ± 0.2 mV; Figure S1). The PS NPs’ test dispersions show similar characteristics to the PS NPs’ stock dispersion (HS: 46.5 ± 0.2 nm; ZP: −24.5 ± 0.3 mV) with a low PdI (0.3). The task of detecting and describing NPls in complex matrices such as soil remains a challenge, as it is currently not possible to do so accurately and reliably [11]. However, it is imperative to urgently address this gap as it is crucial to establish a correlation between the behavior and characteristics of NPls in soil and their detrimental effects on terrestrial organisms. Once NPls enter the soil, they can interact with various soil components, potentially altering their chemical and physical properties, subsequently affecting their reactivity and potential toxicity to organisms [33]. Furthermore, NPls pose a significant challenge in terms of their quantification in environmental matrices. This is primarily due to their small sizes, making them challenging to detect using traditional analytical methods commonly used for larger particles, such as microplastics [34]. Additionally, their diverse shapes, trace-level concentrations, and carbon composition make NPls’ identification and quantification in environmental matrices complex, requiring the use of sensitive, precise, and universal analytical techniques for their detection and quantification [35]. Moreover, the presence of other particles and contaminants in the matrix can interfere with the quantification of NPls, adding to the difficulty of accurately determining their concentration. Furthermore, once detected, the accurate quantification of NPls in complex matrices like soil can be challenging due to matrix heterogeneity, which includes components like organic matter and minerals, as well as the potential for NPls to adsorb soil particles [35]. This complicates the extraction process and measurement of their concentration, emphasizing the need for the development of new analytical methods and techniques capable of selectively detecting and quantifying NPls [34]. Indeed, the field of quantifying NPls in environmental samples still faces significant challenges. To date, only a handful of studies have achieved success in this area [34,35,36,37,38], with low reproducibility rates. Another critical aspect concerning the presence of NPls in soil is the documented phenomenon of plastic particle transport and fragmentation by terrestrial organisms, such as earthworms, collembolans, and mites [10]. These organisms actively move the particles within the soil matrix, and the process of fragmentation can result in the formation of even smaller particles.

### 3.2. Toxicity Tests

#### 3.2.1. Standard Reproduction Test

**Figure 1:**
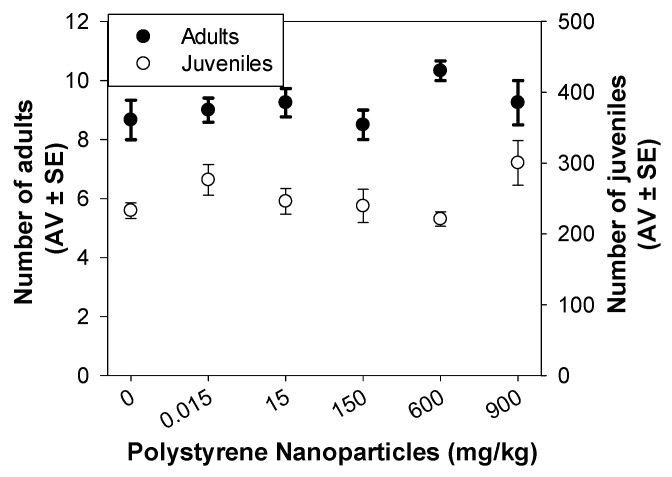
Effects on survival (number of adults) and reproduction (number of juveniles) of *Folsomia candida* after 28 days exposed to polystyrene nanoparticles in LUFA 2.2 soil. Data are expressed as average value (AV) ± standard error (SE).

After 28 d of exposure, the range of PS NPs concentrations tested (0.015 to 900 mg/kg), had no effect on *F. candida* survival (number of adults) or reproduction (number of juveniles) (*p* > 0.05; Figure 1). A representative photo of one replicate captured at the end of the test to allow the counting of *F. candida* organisms using ImageJ software can be found in the Supplementary Material (Figure S2). Previous studies have already shown no effect of PS NPs on the survival and reproduction of *F. candida* (0.015 and 600 mg/kg; 44 nm of diameter; 28 d of exposure [10]) and *E. crypticus* (0.015 to 1500 mg/kg; 49 nm of diameter; 21 d of exposure [11]). According to the review of Gomes et al. (2022) on the ecotoxicological impacts of micro and NPls in terrestrial and aquatic environments, the individual level (where mortality and growth were the most studied endpoints) was the least affected across species, environmental compartments, and polymer types and sizes [8]. Indeed, mortality was never observed, except in one study [39] using the terrestrial species *Eisenia Fetida* exposed to PS flakes (microplastics) at non-environmentally relevant concentrations (5 and 20 g/kg).

#### 3.2.2. Multigenerational Test (F1 to F3)

**Figure 2:**
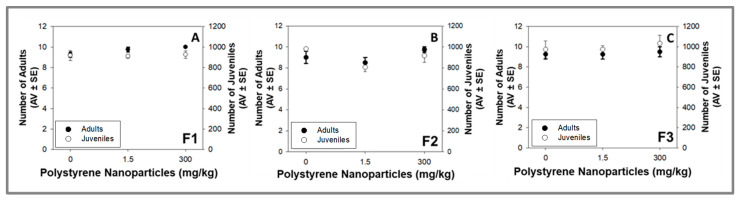
Effects on survival (number of adults) and reproduction (number of juveniles) of *Folsomia candida* exposed to polystyrene nanoparticles in LUFA 2.2 soil for 3 generations (F): (**A**) F1 (parental); (**B**) F2; and (**C**) F3. Each generation was exposed for 28 days. Data are expressed as average value (AV) ± standard error (SE).

PS NPs (1.5 and 300 mg/kg) induced no effects on the organisms’ survival and reproduction along the three generations of *F. candida* (*p* > 0.05; Figure 2A–C). A previous multigenerational study showed that 200 μg/L 70 nm PS NPs decreased the fecundity and prolonged the time to maturation in F1–F3 generations of *B. plicatilis*, with more severe impacts observed in the F3 generation [14]. Multigenerational exposure (F1–F4) of *D. magna* to 200 nm PS NPs induced no effects at 0.1 mg/L and an hormetic response (higher fertility) at 1 mg PS NPs/L for F4 adults [12]. At 10 μg/L, a 35 nm PS NPs exposure of *C. elegans* parental generation (F1) did not induce reproduction toxicity on the subsequent generations (F2–F5) [16]. In the study of Sun et al. (2021), the parental generation was exposed to PS NPs and the subsequent generations were maintained under PS NPs-free conditions [16]. Transgenerational effects of PS NPs were also found for *D. magna*: 13.24 mg/L PS NPs (72 nm) decreased parental reproduction (newborns/brood) and this effect was also found in the subsequent F2 and F3 generations (even not exposed to PS NPs—recovery) [13]. The results obtained in our study, as well as in previous studies [12,14], indicate that the multigenerational effects of PS NPs are dependent on the tested species, PS NPs characteristics and concentrations, and the specific experimental designs employed (exposure occurring for all the tested generations versus parental exposure + subsequent generations of clean media). The found dissimilar effects underscore the importance of increasing the multigenerational studies involving PS NPs and employing different species, particularly those native to terrestrial ecosystems. Notably, the present study is the sole one available that considers a soil medium. Although *C. elegans* is generally classified as a terrestrial organism, soil medium was not utilized in the exposure tests. Moreover, the review of Guimarães et al. (2023) of the available test results from long-term studies showed clear evidence to recommend the implementation of long-term tests in the existing regulatory testing requirements for persistent substances and/or long-lasting effects [9].

### 3.3. Biochemical Markers Analysis

**Figure 3:**
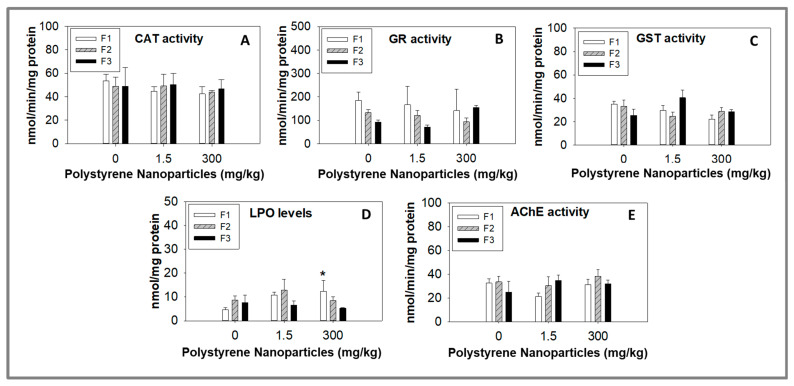
Biochemical responses of juveniles from *Folsomia candida* exposed to polystyrene nanoparticles in LUFA 2.2 soil for 3 generations (F1 to F3), in terms of: (**A**) catalase (CAT) activity; (**B**) glutathione reductase (GR) activity; (**C**) glutathione S-transferases (GST) activity; (**D**) lipid peroxidation (LPO) levels; and (**E**) acetylcholinesterase (AChE) activity. Each generation was exposed for 28 days. Data are expressed as average value (AV) ± standard error (SE). * Significant differences to control (0 mg/kg) (*p* < 0.05).

PS NPs induced no effects on CAT, GR, and GST activities for the juveniles of the three tested generations of *F. candida* (*p* > 0.05; Figure 3A–C), showing that these three enzymes, related by oxidative stress and playing important roles in maintaining cellular homeostasis, were not affected by the tested concentrations (1.5 and 300 mg PS NPs/kg). Despite the fact that no alterations were found on the activities of the tested antioxidant enzymes, oxidative damage through LPO was detected in the juveniles of F1 generation after exposure to 300 mg PS NPs/kg (*p* < 0.006; Figure 3D). However, for the juveniles of the subsequent generations (F2 and F3), an increase in LPO levels was not found (*p* > 0.05; Figure 3D). This finding indicates that the oxidative damage observed in the juveniles of the parental generation did not pass on to the offspring. Instead, it is likely that the descendants developed effective defense mechanisms to prevent the rise of LPO levels, which were not present in the parental generation. A previous study with *F. candida* exposed during 28 d for the same PS NPs used in our study also found an increase in LPO levels (at 0.015 mg PS NPs/kg) with no alterations in the CAT and GST activities [10].

CAT is an antioxidant enzyme that catalyzes the breakdown of hydrogen peroxide (H_2_O_2_) into water (H_2_O) and oxygen (O_2_). This reaction helps to protect cells from oxidative damage caused by H_2_O_2_, a reactive oxygen species (ROS) known to induce LPO [40,41]. GR, another antioxidant enzyme, plays a crucial role in maintaining intracellular levels of glutathione (GSH), an important antioxidant molecule. GR is responsible for reducing oxidized glutathione (GSSG) back to its reduced form (GSH), which can then participate in various antioxidant reactions [42]. GST on the other hand, is an enzyme that conjugates GSH to electrophilic compounds, including reactive intermediates formed during LPO. This reaction helps to detoxify these harmful molecules and protect cells from oxidative damage [43]. Collectively, these enzymes and molecules work together to maintain the balance of oxidative stress and protect cells from the detrimental effects caused by ROS and LPO [44]. LPO, a process in which free radicals target polyunsaturated fatty acids (PUFAs) in cell membranes, leads to the formation of lipid peroxides. These peroxides can further react with other lipids or proteins, causing cellular damage and dysfunction [45]. Antioxidant enzymes like CAT, GR, and GST, along with molecules such as GSH, play a vital role in preventing and mitigating the effects of LPO. However, our study revealed an increase in LPO levels, while the activities of CAT, GR, and GST remained unaffected by PS NPs, which is an intriguing finding that is supported by similar results found in other studies [10,46]. Barreto et al. (2023) showed the potential of these PS NPs to induce LPO (with no CAT and GST activity alteration) for *F. candida* juveniles resultant from organisms exposed for 28 d [10]. Similarly, in *D. magna* exposed for 48 h for PS NPs (100 mg/L) with the same characteristics to the ones used in our study, LPO increased without changes in CAT and GST activities [46]. It is plausible that PS NPs directly affect the LPO pathway, bypassing the antioxidant enzymes we measured. PS NPs can generate ROS directly, initiating LPO independently of antioxidant enzymes [47]. Alternatively, PS NPs may affect other enzymes or pathways involved in LPO, such as cytochrome P450 enzymes or the arachidonic acid cascade [48]. Overall, the relationship between PS NPs exposure, LPO, and antioxidant enzyme activity is complex, depending on various factors, including the specific characteristics of the NPls, the experimental system used, and the endpoints measured. Furthermore, the functioning of crucial enzymes like CAT, GR, or GST may undergo transient modifications which return to baseline levels prior to our measurements. Despite the potential occurrence of such events, they appear insufficient in preventing oxidative damage, as evident from the elevated LPO levels observed. Additional research is necessary to gain a comprehensive understanding of these relationships and their implications for environmental health. The complexities of cellular responses to NPls and oxidative stress make it essential to investigate in detail the specific pathways and interactions involved in this context. The increase in LPO levels observed in the juveniles of F1 (parental generation), but not in the subsequent generations may be attributed to various factors: (1) Adaptive response—subsequent generations may have undergone adaptations or developed detoxification mechanisms in response to stress caused by PS NPs. Organisms have specific regulatory mechanisms that drive changes in gene expression, body morphology, and physiology as a defensive response to stress [41]; (2) Genetic variability—it is possible that the first generation had a higher proportion of individuals with genetic predispositions to LPO, while the subsequent generations had a lower proportion or different combinations of such individuals. Moreover, using descendants from previous generations might have led to increased genetic homogeneity in subsequent generations, which could affect their responses to PS NPs. As a result, selective sweeps could have occurred due to the fixation of favorable alleles, reducing variation in genomic regions near the genes under selection [42,43]. (3) Parental effects—transgenerational epigenetic inheritance or parental provisioning could also play a role [49]. For instance, exposure of the first generation to PS NPs might have induced epigenetic changes in the germ cells that were passed down to subsequent generations, leading to altered gene expression and potentially protective adaptations. Additionally, variations in parental provisioning of antioxidants or other protective compounds between generations could have influenced their susceptibility to oxidative stress [49]. Understanding these factors and their interplay can provide valuable insights into the complex mechanisms driving the observed patterns of LPO levels across generations exposed to PS NPs. Further research is necessary to fully elucidate the underlying processes and implications for environmental health and population dynamics. AChE is an enzyme that plays a pivotal role in regulating the neurotransmitter acetylcholine (ACh) within the nervous system [50]. ACh is a chemical messenger participating in a wide array of physiological processes, including muscle contraction, learning and memory, and regulation of the autonomic nervous system [51]. AChE is found in high concentrations at cholinergic synapses, where it rapidly hydrolyzes ACh released by the presynaptic neuron into acetic acid and choline [51]. Beyond its role in ACh level regulation, AChE is also involved in the detoxification of specific chemicals, making it a frequently utilized biomarker of neurotoxicity in ecotoxicological studies. Although the exact mechanisms of neurotoxicity induced by NPls are not yet fully understood, some studies have suggested that NPls may have neurotoxic effects on organisms by inhibiting the activity of AChE: 1 mg/L (50 nm PS NPs) on *Danio rerio* 120 h exposed [52]; 1 μg/mL (50 nm PS NPs amino-modified) on *Artemia franciscana* 14 d exposed [53]; and 34 μg/L (30 nm PS NPs) on *Aphylla williamsoni* 2 d exposed [54]. For *F. candida*, it seems that PS NPs exposure has no effect on AChE activity, as shown in our study and a previous one [10], showing that if PS NPs induce neurotoxicity for this species, other mechanisms are involved, or AChE activity can be altered earlier and then enzyme activity returns to similar values of the control group. Previous studies already reported some potential mechanisms that can be directly or indirectly involved in the NPls’ neurotoxicity: (a) Activation of immune cells in the brain, leading to the release of pro-inflammatory cytokines that cause inflammation and oxidative stress. This inflammation and oxidative stress can cause damage to neurons and impair their function. (b) Possible direct toxic effects on neurons, possibly due to their small size and ability to penetrate cell membranes and accumulate. This can disrupt cellular processes and lead to cell death. (c) Interference with the normal function of other neurotransmitters (as GABA (Gamma-aminobutyric acid)) in the brain, which can affect mood, cognition, and behavior. (d) Potential induction of epigenetic changes in neurons, altering gene expression and leading to long-term changes in brain function [47,55]. Overall, more research is needed to fully understand the mechanisms of neurotoxicity induced by NPls, especially in the context of long-term exposures. However, it is already recognized that neurotoxicity mechanisms of NPls are dependent on the time of exposure, tested species, and the types/characteristics and concentrations of NPls [56], which can justify the different results found between the available studies.

## 4. Conclusions

The developed multigenerational study, which involved continuous exposure of F1 (parental generation) to F3, showed that PS NPs did not significantly affect the survival and reproduction of *F. candida* over the tested generations. Moreover, the enzymatic responses of the juveniles resultant from the exposed organisms remained unaltered. While LPO levels increased in the juveniles of the parental generation, there was no such increase in the offspring from F2 and F3. Assessing multigenerational effects is crucial for understanding the full impact and potential risks of the contaminants, leading to the development of effective interventions for public and environmental health improvement.

## Data Availability

The data are available on request from the corresponding authors.

## Supplementary Materials

The following supporting information can be downloaded at: https://www.mdpi.com/article/10.3390/toxics11100876/s1, Figure S1: Characteristics of polystyrene nanoparticles stock aqueous dispersion in terms of (**A**) hydrodynamic size (HS) and (**B**) zeta potential (ZP). PdI—polydispersity index; Figure S2: Representative photo of one replicate captured at the end of the test to allow the counting of *Folsomia candida* organisms using ImageJ software.
