# Supplementary material for: New Insights into Nanoplastics Ecotoxicology: Effects of Long-Term Polystyrene Nanoparticles Exposure on Folsomia candida
Source: Toxics. 2023 Oct 22;11(10):876. doi: 10.3390/toxics11100876 (PMC10610651; doi:10.3390/toxics11100876)
Supplement: Supplementary file 1 [file toxics-11-00876-s001.zip › toxics-2649180-supplementary.pdf]

New Insights into Nanoplastics Ecotoxicology: Effects of Long-Term Polystyrene Nanomaterials Exposure on *Folsomia candida*

Angela Barreto[1*], Joana Santos[1], Gonçalo Andrade[1], Matilde Santos[1], Vera L. Maria[1*]

[1] Department of Biology & CESAM, University of Aveiro, 3810-193 Aveiro, Portugal
* Corresponding author: abarreto@ua.pt; vmaria@ua.pt

Supplementary Material

2. Material and Methods

2.3. Test contaminants

PS NPs stock dispersion (10.06% solids in water with 0.1% sodium dodecyl sulfate (SDS) and 0.05% sodium azide (NaN_3)) was acquired from Bangs Laboratories, Inc. According to the supplier, PS NPs had an average diameter of 44 nm, a surface area of 1.299 × 1014 $\mu m^2/g$ and a density of 1.05 g/cm^3. PS NPs stock dispersion was centrifuged prior to the toxicity tests using a Vivaspin® 2 mL ultrafiltration device (Bangs Laboratories, Inc) to remove SDS and NaN_3 present in the dispersion.

2.4.2. Standard reproduction test

At the conclusion of the test, we captured high-quality photos and subsequently choose images with superior definition (Figure S2), focusing on attributes such as sharp edges and absence of blurriness. These selected images were then imported into ImageJ, where we employed the 'multi-point' tool for organism counting. To ensure accuracy, we zoom in to get a better view of the organisms and to differentiate them from the background. We meticulously counted the photographed organisms, section by section. Upon completing this process with the 'multi-point' tool, we obtained the total count of organisms present in each image/replicate/experimental condition.

3. Results and Discussion

3.1. Characterization of polystyrene nanoparticles

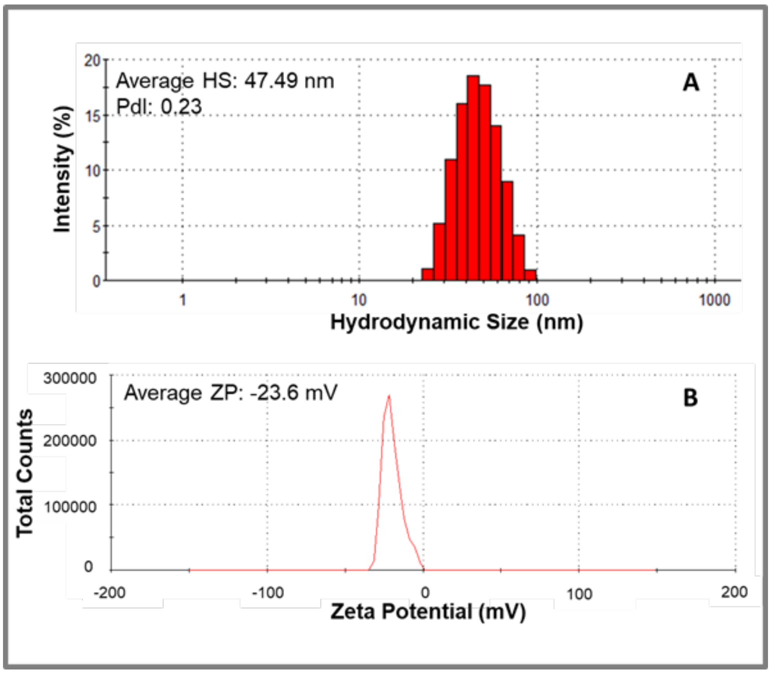

Figure S1. Characteristics of polystyrene nanoparticles stock aqueous dispersion in terms of **(A)** hydrodynamic size (HS) and **(B)** zeta potential (ZP). PdI - polydispersity index.

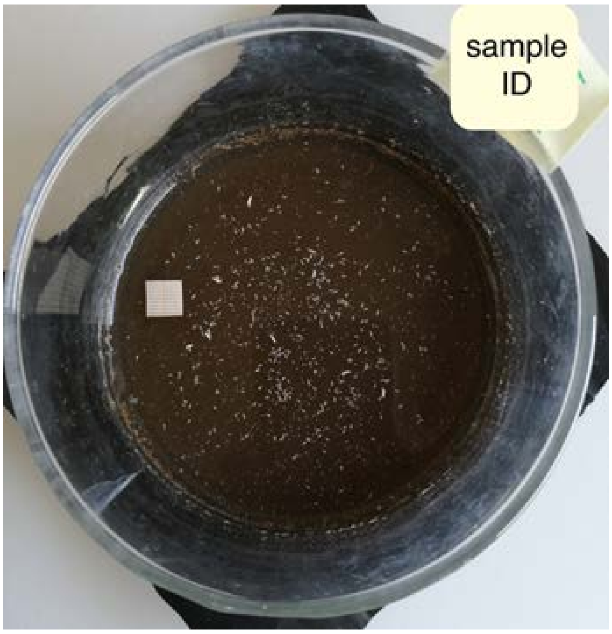

Figure S2. Representative photo of one replicate captured at the end of the test to allow the counting of *Folsomia candida* organisms using ImageJ software.
